# Assessing an aqueous flow cell designed for *in situ* crystal growth under X-ray nanotomography and effects of radiolysis products

**DOI:** 10.1107/S1600577523002783

**Published:** 2023-04-17

**Authors:** Ke Yuan, Vitalii Starchenko, Nikhil Rampal, Fengchang Yang, Xianghui Xiao, Andrew G. Stack

**Affiliations:** a Chemical Sciences, Oak Ridge National Laboratory, Oak Ridge, TN 37831, USA; b Department of Chemical Engineering, Columbia University, NY 10027, USA; c National Synchrotron Light Source II, Brookhaven National Laboratory, Upton, NY 11973, USA; Advanced Photon Source, USA

**Keywords:** X-ray nanotomography, radiolysis, crystal growth, nucleation

## Abstract

Nucleation and growth of minerals has broad implications in the geological, environmental and materials sciences. Recent developments in fast X-ray nanotomography have enabled imaging of crystal growth in solutions *in situ* with a resolution of tens of nanometres, far surpassing optical microscopy. Here, a low-cost, custom-designed aqueous flow cell dedicated to the study of heterogeneous nucleation and growth of minerals in aqueous environments is shown. To gauge the effects of radiation damage from the imaging process on growth reactions, radiation-induced morphological changes of barite crystals (hundreds of nanometres to ∼1 µm) that were pre-deposited on the wall of the flow cell were investigated. Under flowing solution, minor to major crystal dissolution was observed when the tomography scan frequency was increased from every 30 min to every 5 min (with a 1 min scan duration). The production of reactive radicals from X-ray induced water radiolysis and decrease of pH close to the surface of barite are likely responsible for the observed dissolution. The flow cell shown here can possibly be adopted to study a wide range of other chemical reactions in solutions beyond crystal nucleation and growth where the combination of fast flow and fast scan can be used to mitigate the radiation effects.

## Abstract 2

Fast flow and fast imaging can reduce radiation damage in an X-ray nanotomography cell designed for studying mineral nucleation and growth in solutions.

## 1. Introduction

Crystal and amorphous solid growth and dissolution occurring in aqueous environments attracts research applicable to a wide range of fields, such as materials, environmental and geologic sciences (McManus *et al.*, 1998 ▸; Pina & Putnis, 2002 ▸; Fernandez-Martinez *et al.*, 2013 ▸; De Yoreo *et al.*, 2015 ▸; Godinho *et al.*, 2016 ▸; Smeets *et al.*, 2017 ▸). *In situ* observations of nucleation and growth have historically been conducted in flow cells with light-transparent windows (glass or polymer based) under optical microscopy (Hu *et al.*, 2012 ▸). Typically, a reaction vessel, or chamber, is connected to solution pumps where solutions containing the solid’s constituent species are mixed in a separate chamber right before injecting into the reaction chamber. Optical microscopy has been used in nucleation studies by recording the number of nuclei formed as a function of time (Hamm *et al.*, 2014 ▸; Whittaker *et al.*, 2016 ▸). Recent advances in the lab-on-a-chip design have improved our ability to control the mixing processes, where extrinsic factors such as flow patterns and microenvironments around the nuclei can be systematically studied (Poonoosamy *et al.*, 2021 ▸; Haeberle & Zengerle, 2007 ▸; Wang *et al.*, 2017 ▸; Li *et al.*, 2018 ▸). However, a major limitation of optical microscopy is that early stage nanometre-sized nuclei cannot be observed due to its resolution limit. This is especially important when nucleation rates are high relative to growth rates, because optical microscopy underestimates the nuclei density by counting only the larger, micrometre-sized crystals. Another high-resolution *in situ* technique that can image mineral growth and dissolution in liquid is atomic force microscopy (AFM) (Smeets *et al.*, 2015 ▸; Ruiz-Agudo *et al.*, 2015 ▸; Bracco *et al.*, 2012 ▸). The fluid is either flowed parallel to the sample surface or injected near the AFM tip (Bracco *et al.*, 2012 ▸, 2014 ▸), but it is critical to ensure that fluid transport-limited reaction rates are not created. An important factor of using AFM to study heterogeneous nucleation is the potential artifacts induced by the tip–surface interactions (Smith *et al.*, 2003 ▸; Hobbs *et al.*, 2009 ▸). The tip can either remove loosely attached nuclei during scanning or promote crystal growth in the scanned area (Yuan *et al.*, 2019 ▸; Jordan *et al.*, 1999 ▸). Another limitation is the constraints on the slope and height of particles that AFM can measure, *e.g.* any feature on the surface with an aspect ratio steeper than that of the tip will appear pyramidal, which limits characterization of growth rates and morphologies. Liquid-cell transmission electron microscopy (LC-TEM) allows imaging nucleation with a nanometre resolution (Nielsen *et al.*, 2014 ▸; De Yoreo, 2016 ▸). In the liquid cell, a liquid layer of tens to hundreds of nanometres is sandwiched between two electron-transparent membranes. Amorphous-to-crystalline transformation and changes of crystal morphologies have been studied in numerous systems, especially in carbonate minerals (Zhang *et al.*, 2022 ▸; Jin *et al.*, 2021 ▸; Liu *et al.*, 2020 ▸; Zhu, Sushko *et al.*, 2021 ▸). In some cases, changes to solution chemistry due to the formation of radical species induced by the electron beam have been mitigated through low-dose imaging techniques (Moser *et al.*, 2018 ▸; Ambrožič *et al.*, 2019 ▸), and the effects of radiolysis products on dissolution have been quantified for aluminium oxyhydroxides (Liu *et al.*, 2022 ▸), but the effects on solution chemistry and reaction kinetics are unknown in many other systems. Small-angle X-ray scattering (SAXS) and grazing-incidence SAXS (GISAXS) have also been widely used to study nucleation (Dai *et al.*, 2016 ▸; Deng *et al.*, 2019 ▸; Fernandez-Martinez *et al.*, 2013 ▸; Zhu, Li *et al.*, 2021 ▸; Li & Jun, 2019 ▸). In SAXS, nucleation and growth can be performed in an aqueous cell, whereas, in GISAXS, often a static large volume of supersaturated solution is injected into a reactor just prior to measurement without subsequent flow. Particle sizes commonly observed in (GI)SAXS are between 1 and 50 nm, but once crystals quickly grow to micrometre sizes they will not be detected in the small-angle range of the data (Deng *et al.*, 2019 ▸; Dai *et al.*, 2016 ▸; Fernandez-Martinez *et al.*, 2013 ▸). Thus, SAXS experiments are often limited by the nucleation rates they can measure, which exerts constraints on the range of saturation indices and systems that can be explored. Moreover, damage by the X-ray beam to the substrate is of persistent concern as its effect on nucleation (if any) is poorly quantified (Laanait *et al.*, 2015 ▸). Both of these measurements are reciprocal-space measurements, meaning that a scattering model must be fit in order to interpret the data quantitatively.

Here, we introduce the design of an aqueous flow cell used for X-ray nanotomography (XnT) based on the transmission X-ray microscope (TXM) to image the nucleation and growth of barite crystals *in situ* with a field of view of 40 µm × 40 µm and a spatial resolution of 30 nm (Ge *et al.*, 2018 ▸; Yuan *et al.*, 2021 ▸). XnT had been used previously in static solutions to image crystal growth and dendrite formation (Ge *et al.*, 2018 ▸). In the recent work of Yuan *et al.* (2021 ▸), the method was expanded to include several attractions for measuring nucleation and growth, in that it now allows for a flowing solution that maintains a constant chemical potential and quickly removes radiolysis products generated in the solution by the X-ray beam from the imaged area. The fine resolution and comparable field of view with other imaging techniques (*e.g.* AFM) provide new opportunities to quantify heterogeneous nucleation under ambient conditions, especially under high nucleation rates. However, a remaining question is the extent of radiolysis-induced dissolution or other radiation effects. We quantify potential radiation damage by pre-depositing barite crystals on the wall of the capillary tube of the flow cell, and the radiation effects of the X-ray beam on the sample was characterized by reducing the imaging intervals (enhancing the radiation effects), where a 3D tomography was obtained within 1 min under flow conditions. Solution chemistry calculations were also performed to predict potential harmful species that were responsible for the dissolution observed on the crystals. The results demonstrate that, with proper care to avoid radiation-induced artifacts, flow-cell XnT is a valuable addition to a variety of techniques used to study heterogeneous nucleation.

## 2. Methods

### 2.1. Solution and flow cell

**Figure 1:**
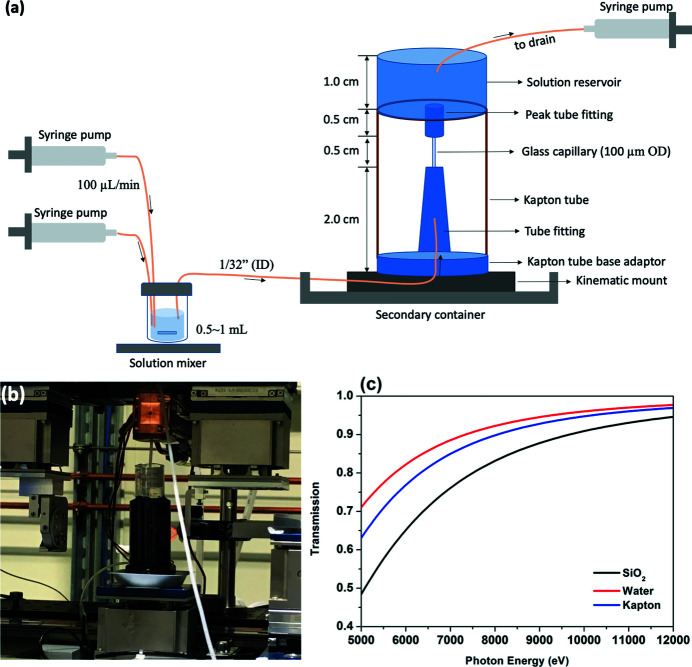
(*a*) Schematic of the liquid flow cell setup used for the X-ray nanotomography experiments. The cell mainly consists of a Kapton supporting tube, a capillary tube and fittings, a solution reservoir, a solution pump and a mixing cell. Materials used in (*a*) are listed in Table S1 of the supporting information. (*b*) Image of the flow cell at beamline 18-ID, NSLS-II. (*c*) X-ray transmission as a function of photon energy for 20 µm quartz, 80 µm-thick water and 127 µm Kapton film.

Solutions containing BaCl_2_ (Sigma-Aldrich), SrCl_2_ (Sigma-Aldrich) and Na_2_SO_4_ (Sigma-Aldrich) were prepared by dissolving the salts into distilled, deionized water (18.2 MΩ cm). The saturation index {SI = log([Ba^2+^][SO_4_^2^]/*K*_sp,barite_ *K*_sp,barite_) = 10^–9.98^} of solutions used for pre-deposition of barite is SI = 2.1, and for imaging radiation effects on barite morphology is SI = 0.0 (*i.e.* barite saturated solution). The flow cell [Figs. 1 ▸(*a*) and 1 ▸(*b*)] consisted of one or two solution reservoirs containing either barium chloride and sodium sulfate, or barium saturated solution, which are loaded inside a syringe pump set at a flow rate of 100 µL min^−1^ (Cole Parmer, Model 270). For supersaturated solutions, a custom solution mixer was included. There was also a glass capillary cell that served as a reaction vessel where radiation-induced dissolution was imaged. The mixer is a sealed glass bottle of maximum volume 2.0 ml with two inlets for the stock solutions, and one outlet that was made of Teflon tubing (1/32-inch inner diameter). To minimize nucleation prior to the portion of the capillary that was imaged, the solution mixer was placed close to the inlet of the capillary. The volume of mixed solution is about 0.5–1 ml with a residence time of 2.5 min inside the mixer under flowing solution. A magnetic stir bar inside the solution mixer mixed the solution by using a stir plate (Fisher Scientific, Model Lab Disk S55). The mixer and attached tubes were thoroughly cleaned in an ultrasonic water bath before use. Rounded quartz capillary tubes (100 µm diameter, Charles Supper Company) with wall thickness of 10 µm were used for the XnT. This thin capillary was used to limit attenuation of the X-ray beam by the silica tube wall [Fig. 1 ▸(*c*)], and it is carefully mounted on the XnT cell where solution flowed from the bottom and drained simultaneously on the top without interrupting the stage rotation [Figs. 1 ▸(*a*) and 1 ▸(*b*)]. All materials used in the flow cell are listed in Table S1 of the supporting information. The cell parts were all available for purchase from suppliers with slight need for post-remodification, which favors disposal of the flow cell after use to eliminate artifacts stemming from nuclei forming on the tubing and mixer walls.

### 2.2. Handling of the small capillary tube and the assembly sequence of the flow cell

The quartz capillary tube has a very fine diameter to reduce the attenuation of the X-ray beam by the tube wall and is susceptible to breaking during the assembly process. The capillary tube was first cut to a length of around 1.5 cm and then glued to the tube fitting adaptor using ep­oxy. The other end of the tube was then connected to a 5 mm-long PEEK tube with crystal bond as the outlet of the cell. The PEEK tube is an essential part that is used to protect the end of the quartz tube from shattering when it is connected to the solution reservoir. The top solution reservoir is then glued to the Kapton cylinder that was cut to a proper length equal to the total height of the tube adaptor, quartz tube and peak tube added together. This Kapton cylinder provides structural support for the solution reservoir assembly, so that the quartz capillary does not bear any weight. A small hole around 2–3 mm is drilled in the middle of the solution reservoir where the PEEK tube passes through and is exposed to the bottom surface of the solution reservoir. The gap between the hole and the peak tube is then sealed using ep­oxy. A concern with using ep­oxy is that as a cured adhesive it may leach volatile species into the growth solution, impacting crystal growth rates. For the dilute, neutral pH solutions used here, along with the minimal contact time of the solutions with the epoxied area, this is likely to have a minimal effect, however. Sealing methods other than the use of ep­oxy would need to be developed in order to seal and connect the quartz tube to cell parts for experiments running under high temperature, in organic and acid solutions, and in any other more reactive solutions.

### 2.3. X-ray nanotomography

XnT was performed at the Full-Field X-ray Imaging beamline (18-ID) of National Synchrotron Light Source II, Brookhaven National Laboratory, USA. The XnT was operated at 9 keV and had a field of view of 40 µm × 40 µm and a resolution of 30 nm. The high adsorption contrast of barite favored the image segmentation of small barite crystals from the background. The sample was imaged *in situ* in the flow cell with an exposure time of 0.05 s for each projection and a total acquisition time of about 1 min for a full 3D tomography scan. The overall transmission of 9 keV X-rays through the cell materials [including 20 µm-thick quartz cell wall (two sides) and 80 µm-thick water] was about 75% [Fig. 1 ▸(*c*)]. To study the radiation effects, each XnT scan was followed by a certain period of rest time during which no beam was exposed to the sample. To nucleate crystals on the inner wall of the capillary tube prior to the XnT measurements, barite growth solutions were flowed through a pristine capillary glass tube. These barite crystals were then used to benchmark the radiation effects. During XnT for these samples, a barite-saturated solution was flowed throughout the experiment. Tomography images were recorded every 30 min for 60 min (three scans), every 10 min for 50 min (five scans) and every 5 min for 20 min (four scans). The XnT data were processed and reconstructed in *TomoPy* (Gürsoy *et al.*, 2014 ▸; Pelt *et al.*, 2016 ▸; De Carlo *et al.*, 2014 ▸). Particle sizes were calculated from the segmented images in *Fiji ImageJ* (Schindelin *et al.*, 2012 ▸; Doube *et al.*, 2010 ▸; Schmid *et al.*, 2010 ▸). A surface resampling value within the *Particle Analysis Toolbox* in *Fiji ImageJ* (Doube *et al.*, 2010 ▸) was used to reduce the noise and remove crystals below the resolution limit when counting the number of particles [see the supporting information of Yuan *et al.* (2021 ▸)].

### 2.4. Radiolysis simulations

Ejection of photons into water excites electrons that can cause the formation of reactive radicals and molecules. The kinetic model used for the simulation of radiolysis of water was adopted from a study by Schneider *et al.* (2014 ▸). Essential species, including 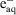 (hydrated electron), H 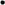 , OH 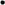 , OH^−^, H^+^, H_2_, O_2_, H_2_O_2_, O 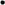 , O_2_^−^, HO_2_, HO_2_^−^, O_3_, O_3_^−^, HO_3_ and O^−^, were taken into account. These were assumed to be uniformly distributed in the system after 1 µs of the photon ejection (Pastina & LaVerne, 2001 ▸). The concentrations of these species were calculated by solving normal differential equations of 79 reactions (Schneider *et al.*, 2014 ▸; see also Table S1 of the supporting information). The primary yield, *G*-value, in units of molecules/100 eV, accounts for the number of given species produced by every 100 eV of adsorbed radiation. For the eight primary radiolysis species of water, their *G*-values are given as the *G*-values for 300 kV electrons in liquid water, based on the precedent by Hill & Smith (1994 ▸). The dose rate (*J*, in Gy s^−1^) defines the adsorbed joules of energy per kilogram per second in the system as estimated by a previous method design for simulating calcite dissolution under X-ray beam (Laanait *et al.*, 2015 ▸). Dose rate, *J*, includes photoelectrons generated by the quartz glass tube, barite crystals and water. However, the number of photoelectrons generated by water are significantly lower than those generated by quartz and barite (10 to 100 times lower), which was not used in the estimation of the final does rate (see supporting information for *J*_water_ calculations),

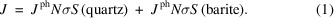

For the quartz portion, *J* ^ph^ is the photon flux density of 10^12^ photons (40 µm)^−2^ s^−1^. The number of quartz molecules per unit area (*N*) illuminated by the X-ray beam is estimated as

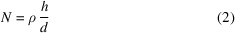

where ρ = 2SiO_2_/33.8 Å^2^ is the number of SiO_2_ per unit area estimated from the surface unit cell of quartz (101), *d* = 3.34 Å is the lattice spacing of quartz (101) along the surface normal direction (Bellucci *et al.*, 2015 ▸), and *h* = 1.2 µm is the depth of the photoelectrons being generated based on the continuous-slowing-down approximation (Laanait *et al.*, 2015 ▸). σ (quartz) = 27.1 cm^2^ g^−1^ is the photoelectron cross-section of quartz at 9 keV (XCOM NIST database), and *S* (quartz) = 19.3 MeV cm^2^ g^−1^ is the stopping powder of quartz at 9 keV (ESTAR NIST database). For barite, we used the following parameters: ρ = 4BaSO_4_/48.423 Å^2^ [barite (001) surface unit cell], *d* = 7.153 Å [lattice spacing of barite (001) along the surface normal direction] (Bellucci *et al.*, 2015 ▸), σ (barite) = 163 cm^2^ g^−1^ and *S* (barite) = 14.33 MeV cm^2^ g^−1^ at 9 keV. The portion of barite surface area illuminated by X-rays is estimated from the tomography image using the projected area of barite on the quartz tube (Fig. S1 of the supporting information). The calculated doses *J*_quartz_ = 1.11 × 10^6^ Gy s^−1^ and *J*_barite_ = 5.24 × 10^5^ Gy s^−1^ yield a total dose rate of *J* = 1.64 × 10^6^ Gy s^−1^. This dose rate is lower compared with a typical TEM (∼10^7^ to 10^8^ Gy s^−1^) (Schneider *et al.*, 2014 ▸) but is higher than that reported for an X-ray reflection interface microscope (∼10^5^ Gy s^−1^) (Laanait *et al.*, 2015 ▸).

## 3. Results

### 3.1. Changes in crystal morphology

**Figure 2:**
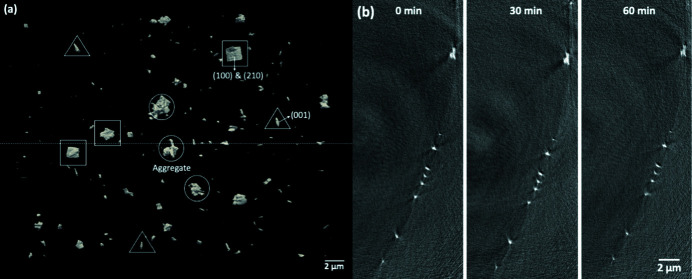
(*a*) 3D view of the prior deposited barite crystals on the inner wall of the quartz capillary tube. Squares, triangles and circles highlight three types of crystals exhibited in different shapes. (*b*) 2D cross-sectional views of the same group of crystals at the dashed line position in (*a*) imaged at 0 min, 30 min and 60 min showing similar morphology.

We performed radiation effects studies on the flow cell by placing a capillary tube filled with barite-saturated solution with previously deposited barite crystals. The 1 min nano­tomography was performed at intervals of 30 min, 10 min and 5 min in order to observe changes occurring to crystals with increasing scan frequency. The substrate was initially covered by about 170 barite crystals of various morphologies and sizes [Fig. 2 ▸(*a*)]. A few large particles (>1 µm) exhibited prismatic forms with visible barite crystallographic facets, such as the barite (001), (210) and (100) crystallographic planes [Fig. 2 ▸(*a*), crystals highlighted by triangles and squares]. Three other relatively large particles did not show any well defined shape but resembled the shape of dendrites formed by aggregation of many small particles, which might indicate multiple nucleation events in the same area [Fig. 2 ▸(*a*), highlighted by circles]. Other small particles of a couple of hundred nanometres in size appeared to be individual single crystals with crystal facets that were obscured because of the finite resolution (30 nm). The cross-sectional views of the same location [dashed line in Fig. 2 ▸(*a*)] at 0 min, 30 min and 60 min showed minor changes in that the smallest features in all the crystals were preserved in all three scans [Fig. 2 ▸(*b*)]. These three sets of data were aligned to correct for minor drift (less than 1 µm) of the imaged areas over 60 min in order to compare crystals at the same location. The 30 min interval scan established the baseline for imaging crystals under flowing conditions, where no crystal dissolution should be observed ideally. Increasing scan frequencies was used next to examine the effects of radiolysis products.

**Figure 3:**
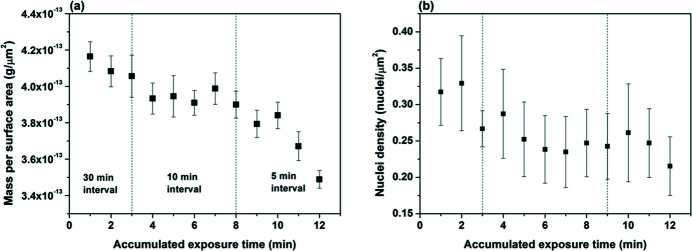
Changes in (*a*) mass per unit surface area of crystals and (*b*) nuclei density as a function of accumulated exposure time to X-rays. Vertical dashed lines indicate the time where scan intervals changed from 30 min to 10 min and from 10 min to 5 min.

Increasing scan frequencies resulted in a reduction of the mass of barite crystals in the beam as well as the nuclei density on the substrate, indicating radiolysis-induced dissolution (Fig. 3 ▸). The mass of the crystals was normalized to their surface areas, where the masses were calculated based on the voxel size and the surface areas of the crystals were obtained by geometric areas assuming cubic-shaped crystals. Mass per surface area of the crystals showed a minor decline (4.0%) when the rest time decreased from 30 min to 10 min [Fig. 3 ▸(*a*)]. A major loss of crystal mass occurred when imaging was taken every 5 min, indicated by where the slope of the curve became more negative, starting at 8 min of accumulated exposure time [Fig. 3 ▸(*a*)]. At the end of the scan, the mass per surface area of the crystals decreased by 16.2% compared with the original value. The nuclei densities showed similar reduction over time (increasing number of scans), where the nuclei density decreased by about 30% at the end of the experiments [Fig. 3 ▸(*b*)]. Error bars in Fig. 3 ▸ were standard deviations obtained by using averaged values from three sets of tomography data generated with increasing threshold values when calculating the voxel sizes or counting the number of crystals [see the supporting information of Yuan *et al.* (2021 ▸)]. The error bars for the nuclei densities appeared to be larger because threshold values were a larger source of uncertainty while counting the number of crystals, where smaller crystals of similar sizes are removed by large threshold values.

**Figure 4:**
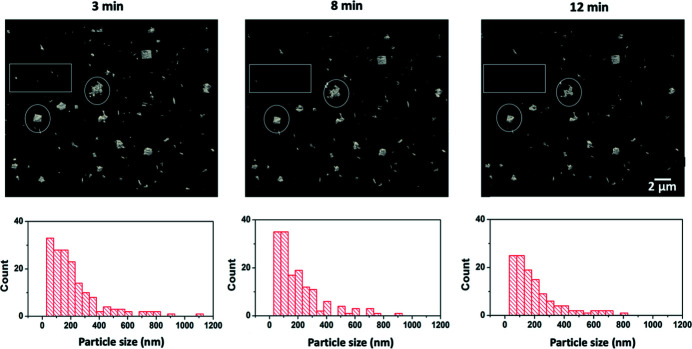
Comparing 3D morphologies of crystals imaged at accumulated exposure times of 3 min, 8 min and 12 min where the scan frequency decreased from every 30 min to every 5 min. Histograms of the corresponding particle size distributions are shown below. Dissolution of larger particles and disappearance of smaller particles are observed.

**Figure 5:**
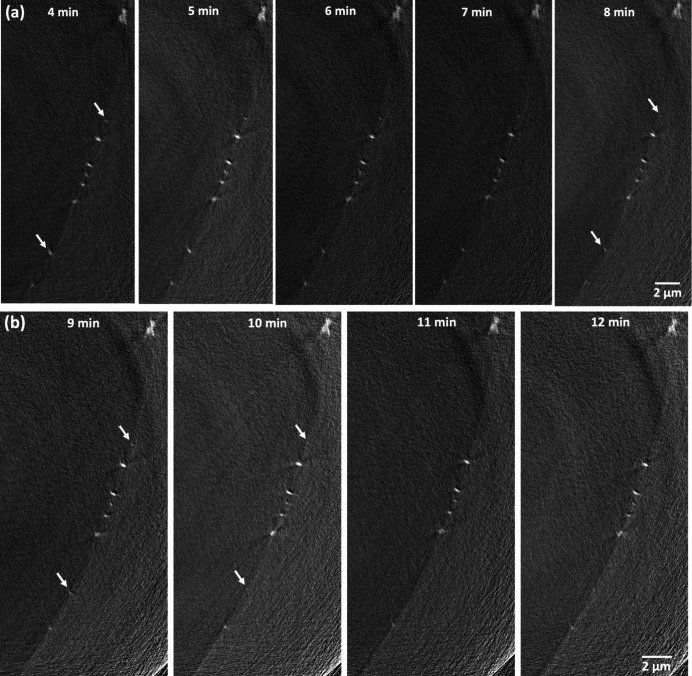
Comparing 2D cross-sectional views of the same location imaged (*a*) every 10 min (accumulated exposure time from 4 min to 8 min) and (*b*) every 5 min (accumulated exposure time from 9 min to 12 min). Arrows indicate dissolution of two small crystals.

Further investigation into the 3D and 2D tomographic data confirmed radiolysis-induced dissolution with increasing number of scans (Figs. 4 ▸ and 5 ▸). Most crystals are less than 400 nm in size at 3 min (end of the 30 min interval scan), with a typical range of 30 nm to 300 nm and few exceeding 1 µm (Fig. 4 ▸, 3 min). The particle size distribution showed a reduction in counts at the range of around 200 nm and an increase at 100 nm (comparing histograms at 3 min and 8 min in Fig. 4 ▸), indicating part of the crystals dissolved into smaller sizes. A few small particles were either reduced in size or disappeared entirely as highlighted by rectangles in Fig. 4 ▸ at 3 min and 8 min. Rounding of corners and edges in a few large crystals is another indication of dissolution (Fig. 4 ▸, two crystals highlighted by circles). The 2D cross-sectional views [Fig. 5 ▸(*a*)] show two small features belonging to two crystals marked by the arrows that appeared smaller over time. Comparing data at 110 min with that at 130 min, all crystals highlighted in the rectangles were dissolved (Fig. 4 ▸). Large crystals showed further reduction in sizes and rounding of corners and edges. This was also shown in the 2D cross-sectional views, where two small features belonging to two crystals indicated by the arrows almost disappeared after 9 min of accumulated exposure time and were not visible at 12 min (Fig. 5 ▸). The particle size distribution showed that the number of particles across all size ranges decreased significantly in the last four scans (Fig. 4 ▸, 8 min and 12 min). Overall, we observed minor dissolution in the first 7 min of scans (scan intervals of 30 min and 10 min), and major size reduction and dissolution were found when the sample was imaged every 5 min.

### 3.2. Radiolysis products in aqueous solutions

**Figure 6:**
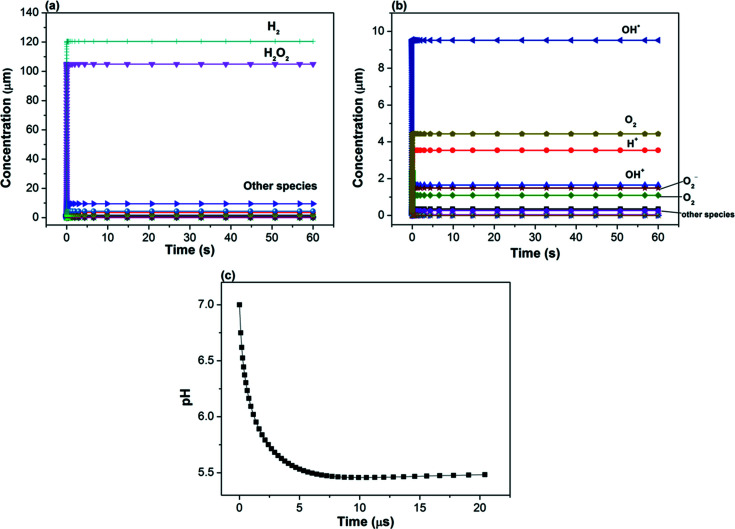
(*a*) Calculated radical and molecular species over 60 s. (*b*) Zoom-in view showing evolution of other species of low concentrations over time. (*c*) Evolution of pH over time in the first 20 µs.

Illuminating water and solids with ionizing radiation, such as X-ray photons, ejects energetic primary electrons which leads to the formation of secondary electrons that can produce a series of electron–hole pairs, damaging reactive species in solution (*e.g.* hydrated electrons, hydroxyl radicals), and reactive molecular species (*e.g.* OH^−^, H^+^, H_2_ and H_2_O_2_) that may migrate to the solid–water interface (Loh *et al.*, 2020 ▸; Hill & Smith, 1994 ▸; Conroy *et al.*, 2017 ▸). These processes occur over a very short period (µs) and can significantly modify the solution chemistry, such as the local pH near mineral–water interfaces, that may impact the supersaturation level of the growth solutions used for crystal nucleation. The radiolysis simulation described above was performed for 60 s (scan time for collecting one tomography data set) and the results showed that H_2_ and H_2_O_2_ had much higher steady state concentrations (120 and 105 µ*M*, respectively) than other species [<10 µ*M*, Figs. 6 ▸(*a*) and 6 ▸(*b*)]. In addition to H_2_ and H_2_O_2,_ which are at the ∼100 µ*M* level, OH 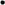 , O_2_ and H^+^ had relatively high concentrations above ∼3 µ*M*. The pH of the solution decreased from 7 to around 5.5 because of the production of H^+^ [Fig. 6 ▸(*c*)]. Concentrations of all species reached equilibrium states in less than 10 µs.

## 4. Discussion

### 4.1. Radiation effects on the crystal

Right after exposure of an X-ray or electron beam to water, gas bubbles were observed in static solution in the current experiments (data not shown). Previous studies have shown that this is due to the generation of H_2_, O_2_ and decay products of H_2_O_2_ (Fig. 6 ▸) (Grogan *et al.*, 2014 ▸). Therefore, flowing solution is needed in order to quickly remove these gases. The flow rate in our experiment is 100 µL min^−1^, as controlled by the push–pull syringe pump (Fig. 1 ▸). Assuming a round tube of diameter 80 µm and length 1.5 cm, the total amount of solution inside the tube will be replenished every 0.05 s. The actual flow rate inside the tube may be slower because of factors such as distance (2.5 m) between pump and flow cell, imperfect connections between capillary tube and cell parts, growth of crystals thereby narrowing the cell volume, and pressure from the mass of solution in the reservoir on top of the quartz tube. However, even assuming a ten-times-slower flow rate of 10 µL min^−1^, the solution inside the capillary tube will be refilled in less than 1 s, which is sufficient to remove gas products (H_2_ and O_2_). It may be possible to use capillaries of larger diameters in the future to reduce pressure and facilitate flow. The key factor is to limit the wall thickness of the capillary tube, because it significantly attenuates the X-ray beam [Fig. 1 ▸(*c*)].

The dissolution rate of barite is mainly a function of the accumulated X-ray dose under the 5 min interval scans; however, longer scan intervals (30 min) may have allowed the surface damaged by radiolysis products to recrystallize. Previous studies by Kang *et al.* (2022 ▸) showed that Ba on barite surfaces was constantly exchanging with solution Ba^2+^ under near equilibrium conditions (equilibrium recrystallization) by using isotope substitution techniques to trace the forward and backward dissolution/growth rates. This is consistent with prior computational work showing facile rates of ion attachment and detachment to barite surfaces (Stack *et al.*, 2012 ▸). The recrystallization process may be important to heal the high-energy surface sites (kinks) created by radiolysis products. Furthermore, X-ray nanotomography study on the dissolution of single calcite crystals showed that the dissolution rate of calcite increased with time. Accumulated dissolution time exposed more reactive surface sites on calcite. We hypothesize that the longer interval between scans leads to the high-energy surface sites around etch pits on barite surfaces to recrystallize, which would result in minimal dissolution in the following scans. Following this hypothesis, the shorter interval might create more reactive sites over time and promote the dissolution of barite because there was limited time to heal the surface.

The nearly instantaneous equilibrium of radicals and molecules generated by water radiolysis within the capillary tube limited the radiation damage primarily to the 1 min scan period when the beam is actively on the sample. Other accumulated dose was from the white-field data collection time at the beginning of the scan and any possible rest time between the end of scan and closure of the X-ray shutter (few seconds). When using the last four data points of the 5 min interval scans [Fig. 3 ▸(*a*)] to calculate the dissolution rate of barite (assuming the reaction occurred in 4 min, corresponding to four 1 min tomography scans), we obtained a radiolysis-induced dissolution rate of 6.4 × 10^−7^ mol m^−2^ s^−1^. When using the 30 min and 10 min rest-time data to derive the dissolution rate, we obtained a rate of 2.9 × 10^−7^ mol m^−2^ s^−1^. Both rates are larger than the barite dissolution rates (10^−8^ mol m^−2^ s^−1^) reported previously at pH = 2–10 in undersaturated solutions of SI < 0.0 (Zhen-Wu *et al.*, 2016 ▸). To understand this process, we evaluate the radiolysis products individually. The first species that may impact barite growth and dissolution is H^+^. Ruiz-Agudo *et al.* (2015 ▸) showed that the growth and nucleation rates measured on barite surfaces by AFM had no major changes between pH 3 to 9, whereas the measured rates started to increase when pH was above 10. Barite dissolution rates measured experimentally by Zhen-Wu *et al.* (2016 ▸) showed that the measured rate constants increased with decrease of pH from 9 to 2. Our simulations indicated that the solution pH decreased from 7 to 5.5 within 10 µs after illumination of the X-ray beam (assuming static solution). It seems that the change of pH likely has a minor impact on the observed dissolution of barite. However, the pH close to the surface of the barite–water interface may be significantly lower than 5.5 when the beam was on the sample. When we compare our above radiolysis-induced dissolution to the pH-dependent dissolution rate measured by Zhen-Wu *et al.* (2016 ▸), we found that our rate, derived from the 30 min and 10 min interval scans (2.9 × 10^−7^ mol m^−2^ s^−1^), is close to the rate measured at pH = 4.2 by Zhen-Wu *et al.* (2016 ▸) – see Fig. S2 of the supporting information. In contrast, our rate obtained from the 5 min interval scan (6.4 × 10^−7^ mol m^−2^ s^−1^) is equivalent to a pH value of −7.6, indicating that the local environment at the interface is likely very different from that of the bulk solution. Another major radiolysis product of relatively high concentration is H_2_O_2_ [Fig. 6 ▸(*a*)]. The decomposition of hydrogen peroxide can produce oxidants, such as O_2_ and OH 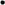 (Mlasi *et al.*, 2015 ▸). Hydrogen peroxide has been used to process ores containing redox-sensitive metals, such as sulfide and uranium ores, where these minerals dissolve through oxidative dissolution reactions (Adebayo *et al.*, 2003 ▸). Ba^2+^ is not a redox-sensitive metal under ambient conditions and sulfate groups in BaSO_4_ cannot be further oxidized by the decomposition products of H_2_O_2_. Other than pH and H_2_O_2_, the production of other short-lived reactive radicals/molecules may also be responsible for the dissolution of barite; however, the reaction mechanisms of these species on barite dissolution remain unclear. In addition to chemical dissolution, the movement of any transient gas bubbles (H_2_ and O_2_) along the tube wall, if formed, may remove some of the loosely attached crystals physically. Overall, it is most likely that the reactive radicals formed near the barite–water interface and a decrease of local pH are responsible for the observed dissolution (Conroy *et al.*, 2017 ▸).

## 5. Summary

We demonstrate an economical, liquid flow cell designed for imaging crystal growth under X-ray nanotomography. All parts of the flow cell are available for purchase from existing suppliers with minor amount of post-processing effort required. We observed radiolysis-induced dissolution on crystal inside the cell. However, the radiation damage can be readily mitigated by using a fast flow rate combined with fast data collection time (60 s) and appropriate resting time between scans (>5 min). The water radiolysis simulations indicated a decrease of solution pH by 1.5 units and the generation of harmful radical/molecular species could potentially lead to the dissolution of barite. Future improvements to the cell may include the design of ep­oxy-free adaptors to connect the capillary tube with the cell body, such that it can tolerate high temperature and pressure, which may be required to simulate harsh environments under subsurface conditions.

## Supplementary Material

List of materials used for the flow cell; estimation of does rate from water; estimated pH change from the observed dissolution rates. DOI: 10.1107/S1600577523002783/vy5005sup1.pdf
