# Supplementary material for: Assessing an aqueous flow cell designed for in situ crystal growth under X-ray nanotomography and effects of radiolysis products
Source: J Synchrotron Radiat. 2023 Apr 17;30(Pt 3):634–42. doi: 10.1107/S1600577523002783 (PMC10161885; doi:10.1107/S1600577523002783)
Supplement: Supplementary file 1 [file s-30-00634-sup1.pdf]

JOURNAL OF SYNCHROTRON RADIATION

**Volume 30 (2023)**

**Supporting information for article:**

# Assessing an aqueous flow cell designed for *in-situ* crystal growth under X-ray nanotomography and effects of radiolysis products

**Ke Yuan, Vitalii Starchenko, Nikhil Rampal, Fengchang Yang, Xianghui Xiao and Andrew G. Stack**

**Table S1** Materials used for the construction of the flow cell.

| Materials | Brand |
|---|---|
| Peak tube | Cole-Parmer (1/32" ID) |
| Kinematic mount | MKS Newport, M-BK-1A-T |
| Peak tube adaptor | Cole-Parmer |
| Teflon tube | McMaster-Carr (1/32" ID) |
| Quartz glass capillary | Charles Supper Company, 100 µm diameter round quartz tube, 10 µm wall thickness |
| Kapton film | McMaster-Carr (127 µm thickness) |

### S1. Estimation of does rate from water

$\rho$ = 0.012 water/$Å^2$ is the number of water molecules per unit area estimated from 0.033 water/$Å^3$ (number density of water) integrated over a thickness (d) of Ro-o, which is the liquid water O-O distance of 2.79 Å obtained from a typical neutron radial distribution function data (ref). $h$ = 1.2 µm is the depth of the photoelectrons being generated based on the continuous-slowing-down approximation. $\sigma(water)$ = 7.43 $cm^2/g$ (XCOM NIST database) is the photoelectron cross-section of water and S (water) = 24.48 MeV $cm^2/g$ (ESTAR NIST database) is the stopping powder of water at 9 keV. Dose rate *J (water)* = $2.81 \times 10^4$ Gy/s.

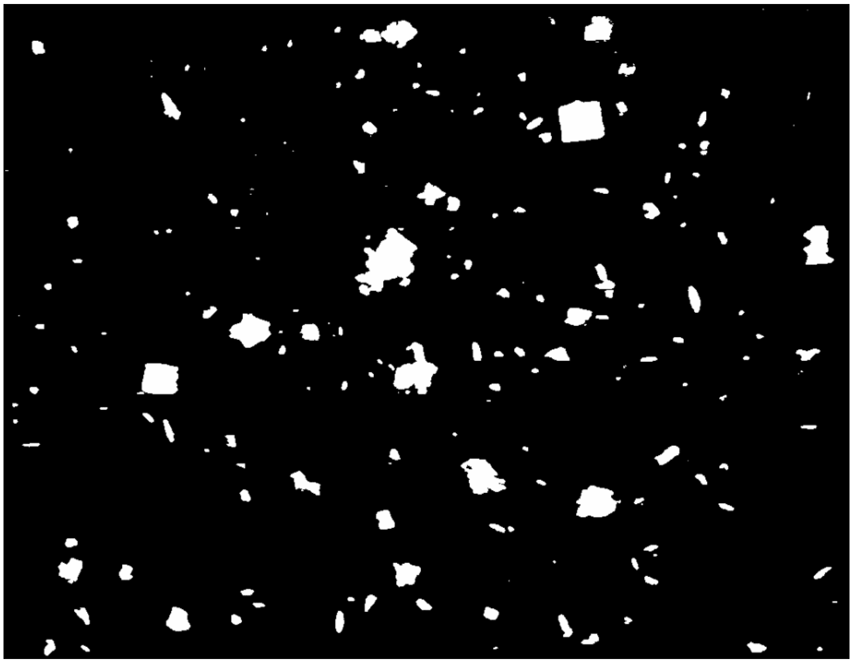

**Figure S1** Estimation of surface area of barite illuminated by the X-ray beam. The binary image shown in Fig 2a was projected onto the quartz surface of an area of 827 $\mu m^2$, yielding a 4.16% of the total field of view covered by barite.

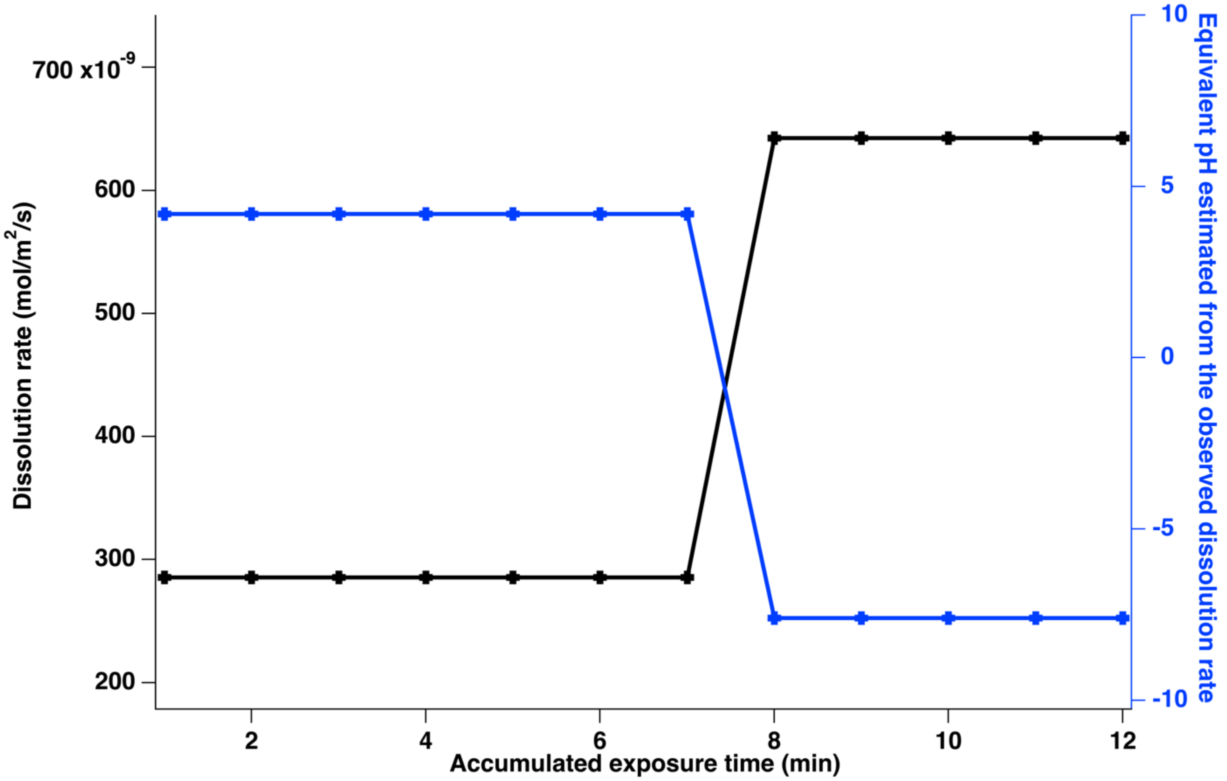


**Figure S2** Dissolution rates and associated pHs as a function of the accumulated exposure time. pH values were calculated based on the observed dissolution rates according to ref [50]. The barite dissolution rate r = k($\Omega^{0.2}$-1), where r is the dissolution rate in mol/m$^2$/s, k is the rate constant, and Ω is the saturation state in $[Ba^{2+}][SO_4^{2}]/K_{sp,barite.}$ For simple estimation, we assume the solution saturation state close to the barite surface Ω = 0 in our experiments, therefore, the calculated dissolution rate r will equal to the rate constant k. Two different dissolution rates were estimated. Dissolution rate 1 was calculated based on data from 1 min to 8 min and the dissolution rate 2 was calculated based on data from 8 min to 12 min in Fig. 3a.
